# Application of a plain abdominal radiograph transition zone (PARTZ) in Hirschsprung's disease

**DOI:** 10.1186/1471-2431-7-5

**Published:** 2007-01-27

**Authors:** Akshay Pratap, Devendra K Gupta, Awadhesh Tiwari, Arvind K Sinha, Nisha Bhatta, Satyendra N Singh, Chandra S Agrawal, Anand Kumar, Shailesh Adhikary

**Affiliations:** 1 Division of Pediatric Surgery, B.P. Koirala Institute of Health Sciences, Dharan, Nepal; 2 Department of Pediatric Surgery, All India Institute of Medical Sciences, New Delhi, India; 3 Department of Radiology, B.P. Koirala Institute of Health Sciences, Dharan, Nepal; 4 Department of Pathology, B.P. Koirala Institute of Health Sciences Dharan, Nepal; 5 Department of Pediatrics, B.P. Koirala Institute of Health Sciences Dharan, Nepal; 6 Department of Anesthesia, B.P. Koirala Institute of Health Sciences, Dharan, Nepal

## Abstract

**Background:**

A standard contrast enema for Hirschsprung's disease can sometimes be inconclusive in delineating a transition zone especially in neonates and infants. The aim of this study was to determine the utility and diagnostic accuracy of a plain abdominal radiograph transition zone (PARTZ) in predicting the level of aganglionosis.

**Methods:**

A prospective observational study of neonates and infants with biopsy proven Hirschsprung's disease was carried out from March 2004 through March 2006. All patients underwent a plain abdominal radiograph and a contrast enema followed by a rectal biopsy. The transition zone on a plain radiograph (PARTZ) and contrast enema (CETZ) were compared with operative and pathology reports. Results were analyzed by chi square test and expressed as their p values and 95% confidence intervals.

**Results:**

PARTZ and CETZ suggestive of Hirschsprung's disease was seen in 24(89%) and 18(67%) patients respectively. The PARTZ and CETZ matched with the pathologic level of transition zone in 22(92%) and 13(72%) patients, p = 0.001, 95% CI (-1.87 to -0.79). In the 9 (33%) patients in whom contrast enema failed to reveal a transition zone, PARTZ was seen in 6/9(66%) patients and correlated with the pathological level of aganglionosis in 4/6(67%) patients, p = 0.001 95% CI (-1.87 to -0.79). The overall accuracy of PARTZ and CETZ was 96% and 84% respectively, p = 0.008, 95% CI (-6.09 to -3.6).

**Conclusion:**

A plain abdominal radiographic transition zone is reliable in predicting the level of transition zone in cases of inconclusive contrast enema. It may be particularly helpful developing countries where laparoscopic techniques are not available to accurately identify the transition zone.

## Background

Hirschsprung's Disease (HD) is a common cause of pediatric intestinal obstruction [1]. A funnel shaped transition zone on a contrast enema (CETZ) at the junction of aganglionic and ganglionic gut is considered a hallmark for its diagnosis [2,3]. In recent years, primary pull-through procedures, especially the transanal pull through, has become popular for the treatment of rectosigmoid Hirschsprung's disease [4,5]. Knowledge of the extent of aganglionic bowel on contrast enema is important for preoperative planning of trans-anal surgery [6]. Although CETZ remains a reliable diagnostic sign, some investigators have found its accuracy to range between 80% to 94%, with a 20% false negative result in neonates and infants [7,8]. The usefulness and accuracy of a transition zone visible on a plain abdominal radiograph (PARTZ) has not yet been evaluated. We therefore conducted a prospective study to investigate whether PARTZ could reliably predict the level of transition zone.

## Methods

From March 2004 to February 2006, neonates and infants with clinical suspicion of Hirschsprung's disease were enrolled in this prospective observational study that was approved by the Ethics committee of B.P Koirala Institute of Health Sciences, Nepal.

### Patients inclusion criteria

1) Delayed passage of meconium (beyond 48 hours for a full term neonate and beyond 72 hours for a preterm neonate).

2) Patients with defecation problems since birth and abdominal distension.

### Patients exclusion criterion

1) The modified Bell staging criteria [9] in which a composite of clinical signs and symptoms (eg, abdominal distention, bloody stools, or hypotension), biochemical parameters (eg, thrombocytopenia or neutropenia), and radiographic signs (eg, pneumatosis or pneumoperitoneum) was used to grade the severity of NEC.

2. Abdominal radiograph showing multiple air fluid levels.

Written and verbal informed consent was taken from the patients who satisfied the inclusion criteria to undergo further investigations. Data on gestational age and first passage of meconium after birth were collected. Prior to per rectal examination, all patients underwent a plain abdominal radiograph and a contrast enema.

### Plain abdominal radiograph transition zone

A plain abdominal erect radiograph was taken to visualize tapering and abrupt cutoff of left colon gas shadow above the pelvis, which indicated the level of PARTZ. All plain abdominal radiographs were read by the same radiologist (A.T).

### Contrast enema

Pediatric radiologists performed the contrast enema in a routine manner using standard CE techniques. Dilute barium sulfate was administered rectally using a # 6 infant feeding tube placed just within the rectum. No balloon catheters were used. All CE images were read by the same pediatric radiologist (A.T). The classical finding of a transition zone [2] (CETZ) was considered being a positive result.

### Rectal biopsy

The final diagnosis of HD was made by the absence of ganglion cells in a full thickness biopsy (FTB). Biopsy specimens were obtained at 2 cm above the dental line, posteriorly. These specimens were examined for ganglion cells with a hematoxylin-eosin staining and acetylcholinesterase activity was determined as previously described by Karnovsky and Roots [10]. A biopsy was considered to be positive when the acetylcholinesterase activity was elevated in combination with an absence of ganglion cells. An experienced histopathologist with specific interest and expertise evaluated the biopsies for HD (A.S).

The decision to perform a single stage transanal pull through was determined by the patient's general condition, and the presence of a rectosigmoid or midsigmoid transition zone on a plain abdominal radiograph or CE. Transanal pull through was performed using the operative technique described previously [4]. Following the surgery, the location of PARTZ and CETZ was compared with pathology reports documenting the level of aganglionosis. For the purpose of this comparison, the bowel was divided into 9 segments: rectosigmoid, midsigmoid colon, descending colon, splenic flexure, transverse colon, hepatic flexure, ascending colon, and cecum/small bowel (total colon). The PARTZ and CETZ were determined to be concordant if located in the same/adjoining bowel segment as the pathologic level of aganglionosis or discordant if they were separated from the level of aganglionosis by at least one intervening bowel segment.

### Statistical analysis

We tested for the concordance between the radiographic transition zone (PARTZ and CETZ) and the pathologic extent of aganglionic bowel using chi square test with a 95% confidence interval. In all analyses, *P *values <0.05 were considered to be statistically significant. Statistical analyses were performed using the SPSS software (version 12.01, Chicago, IL, USA).

## Results

Twenty seven patients (20 neonates and 7 infants) were included in the study, Table 1. A total of 24 transanal pull through procedures were performed. Data of the results of the tests and their concordance with pathological level of aganglionosis are shown in Table 2.

### Correlation of plain abdominal radiograph transition zone and its pathologic location

**Figure 1:**
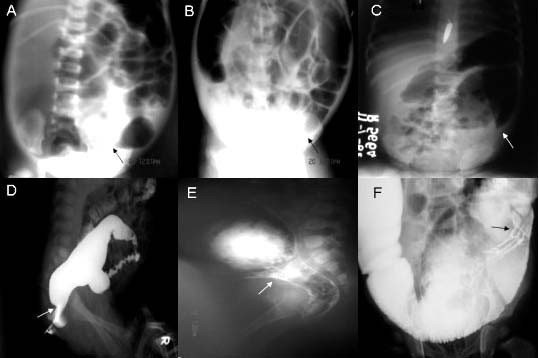
A: Plain abdominal radiograph showing a PARTZ at rectosigmoid, arrow. B: Plain abdominal radiograph showing a PARTZ at midsigmoid, arrow. C: Plain abdominal radiograph showing a PARTZ at descending colon, arrow. D: Contrast enema showing a CETZ at rectosigmoid, arrow. E: Contrast enema showing a CETZ at midsigmoid, arrow. F: Contrast enema showing a CETZ at descending colon, arrow.

A plain abdominal radiograph showed tapering of left colon gas with an abrupt cutoff indicative of a transition zone (PARTZ) in 24/27 (89%) patients. The PARTZ was located at rectosigmoid in 21(88%), midsigmoid in 2(8%) and at descending colon in 1(4%), Fig 1A,1B,1C respectively. Of the 24 patients with a PARTZ, 22 (92%) had a matching level of aganglionosis [p = 0.00, 95% CI (-4.4 to -3.93)]

### Correlation of contrast enema transition zone and its pathologic location

A CETZ was seen in 18/27(67%) patients. The CETZ was located at rectosigmoid in 14(78%), midsigmoid in 2(11%) and descending colon in 2(11%), Fig 1D,1E,1F. Of the 18 patients with CETZ, 13 (72%) had a matching level of aganglionosis [p = 0.6, 95% CI (-0.85 to 0.52)].

### Inconclusive contrast enema versus PARTZ

Contrast enema failed to reveal a transition zone in 9/27(33%) patients. A PARTZ was seen in 6/9(66%) of these patients. PARTZ correlated with the pathological level of aganglionosis in 4/6(67%) patients, p = 0.001 95% CI (-1.87 to -0.79).

### Comparison of PARTZ with CETZ

The overall accuracy of PARTZ and CETZ concordant to the pathological level of aganglionosis was 92% and 72% respectively, p = 0.008, 95% CI (-6.09 to -3.6).

## Discussions and Conclusion

The hallmark radiological feature of HD is the presence of a transition zone on a contrast enema (CETZ) [2]. One of the requisites for successful pullthrough surgery for Hirschsprung's disease is identification of the transition zone, for which a contrast enema is relied upon. Although CETZ remains the most accurate diagnostic sign for Hirschsprung's disease, it is not specific enough to delineate the transition zone in neonates and infants [7,8,11]. Other radiographic signs to improve the diagnostic yield, including delayed and abnormal contractions of distal aganglionic segment also appear to be of limited value [2,12]. There has been a recent trend in the use of preoperative endoscopic marking of the transition zone, and laparoscopy-assisted suction colonic biopsy (SCBx) to provide accurate identification of the transition zone[13,14]. However, these investigations are not available in most developing countries. A plain abdominal radiograph, which is routinely done to evaluate any intestinal obstruction including HD, may provide more information than just the diagnosis. Its utility to locate a plain abdominal radiograph transition zone (PARTZ), especially when CETZ is inconclusive, has not been previously studied. In this study, PARTZ was clearly seen in 89% of the patients, which accurately corresponded with the pathological level of aganglionosis in 92% of the patients undergoing a pull through procedure. A CETZ on the other hand was conclusive in only 67% of the patients. Importantly, for this subset of patients with inconclusive CETZ (9 patients), a PARTZ accurately correlated with the pathological level of aganglionosis in 4(67%) patients. A false negative or inconclusive CE may be attributable to technical factors, too much or forceful instillation of contrast, a small caliber of neonatal bowel, prior colonic washouts or a long segment disease[15,14]. These factors may obliterate the transition zone. On the other hand, visualization of PARTZ relies on the physiological tapering of bowel gas in the transition zone above the distal collapsed gasless aganglionic segment, which is left undisturbed by avoiding instillation of any contrast in the rectum. Although in cases with inconclusive CE studies, retention of barium seen on radiographs obtained 24 hours after a barium enema is considered suggestive of Hirschsprung's disease [2], the level of transition zone remains uncertain unless laparoscopy is employed [14]. If facilities of laparoscopy are not available, an umbilical incision provides an excellent, safe, and versatile alternative to laparoscopy or other abdominal incisions [17]. In conclusion, our study underscores the importance of combining the information of a transition zone on a plain abdominal radiograph and contrast enema to decide the surgical approach for the correction of Hirschsprung's in developing countries where laparoscopic facilities are not available. The small incidence of discordance between anticipated level of aganglionosis and operative findings should be recognized, particularly when planning a one-stage transanal pull-through.

## List of abbreviations used

1. HD: Hirschsprung's disease

2. CE: contrast enema

3. CETZ: contrast enema transition zone

4. PARTZ: Plain abdominal radiograph transition zone

5. FTRB: Full thickness rectal biopsy

6. PLAG: Pathological level of aganglionosis

## Competing interests

The author(s) declare that they have no competing interests.

## Authors' contributions

AP and DKG have been involved in the design of the study, acquisition, analysis and interpretation of data. AT and AKS performed the radiological and pathological analysis.

SNS provided anesthesia support for surgery. NB, CSA and SA helped to perform statistical analysis, and revising the manuscript critically.

All authors read and approved the final manuscript.

## Pre-publication history

The pre-publication history for this paper can be accessed here:



## Figures and Tables

**Table 1:** Patient characteristics

Patient characteristics	Total group N = 27
Neonates (< 1 month)	20
Infants (1 to12 months)	7
Prematurity	4
Delayed meconium passage>24 hours	20
PARTZ visible	24
CETZ visible	18
FTRB confirming Hirschsprung's disease	27
Transanal pull through performed	24

FTRB, Full thickness rectal biopsy; PARTZ, Plain abdominal transition zone; CETZ, Contrast enema transition zone.

**Table 2:** Data comparing the correlation results of PARTZ and CETZ with pathological level of aganglionosis

Correlation between Parameters	p value	95% Confidence interval
PARTZ vs. CETZ	*0.008	-6.09 to -3.6
PARTZ vs. PLAG	*0.00	-4.4 to -3.93
CETZ vs. PLAG	0.6	-0.85 to 0.52
PARTZ vs. ICCE	*0.001	-1.87 to -0.79

PARTZ, Plain abdominal transition zone; CETZ, Contrast enema transition zone; PLAG, Pathological level of aganglionosis; ICCE, Inconclusive contrast enema. * p < 0.05 significant
